# Mississippi Valley Dental Society

**Published:** 1896-05

**Authors:** 


### Mississippi Valley Dental Society.

The fifty-second annual meeting of the Mississippi Valley Dental Society was held on the 15th and 16th of April, and manifested about its usual vigor and interest in professional matters. The programme prepared by the Executive Committee was a very good one indeed. Most of the subjects presented were in reference to new things or new phases of things that have been used for a time. Among the dental appliances and processes exhibited were the "Hot Air Cavity Dryer," by Dr. Wassell, of Chicago; "Collection of Experimental Root Fillings," by Dr. Harlan, of Chicago; "A Water Rheostat for Cataphoresis," by Dr. Wm. H. Hersh, Piqua, Ohio; "The Jennings' Electrical Cataphoric Obtunder," by Dr. Callahan, of Cincinnati; "Atmospheric Dentures, Upper and Lower," by Dr. Ames, of Chicago.

Papers on the following subjects were read and discussed as fully as the time would allow: "A Suggestion on the Origin of some Cases of Pyorrhœa Alveolaris," by Dr. Matlack, Cincinnati; "Roentgen X Rays," by Dr. Sage, Cincinnati; "Ground Porcelain Inlays," by Dr. Harlan, of Chicago; "A Suggestion," by Dr. Bethel, of Kent, Ohio; "Progressive Calcification," by Dr. Cravens, Indianapolis; "Treatment of Sensitive Dentine by Cataphoresis," by Dr. Custer, Dayton, Ohio; with demonstrations.

These papers will be published in the DENTAL REGISTER with a synopsis of the discussions had upon those which were discussed.

It was an excellent programme, but contained altogether too much matter for the time allotted to the sessions. In the discussions justice was not done to a single one of the papers read. Almost every one who attempted to discuss a paper was on short time, and many were prepared for discussion who had not the opportunity. Nevertheless, the meeting was a good one, its social features very pleasant, and on the whole the Society demonstrated the fact that it was capable of yet doing grand work for the profession.

The officers for the ensuing year are Dr. J. E. Cravens, Indianapolis, President; Dr. H. T. Smith, Cincinnati, Secretary; Dr. Frank Hunter, Cincinnati, Treasurer.

The election of officers occupied only about ten minutes. The Mississippi Valley has in all respects only a minimum of routine business. For this purpose not over thirty minutes was employed during the whole meeting. Long live the Mississippi Valley Society of Dental Surgeons.

